# Analysis of grip specificity on force production in grapplers and its effect on bilateral deficit grip specificity and bilateral deficit in force production among grapplers

**DOI:** 10.3389/fspor.2023.1190369

**Published:** 2023-09-25

**Authors:** Raquel Escobar-Molina, Miguel Cuevas-Laguna, Ignacio J. Chirosa-Ríos, María Merino-Fernández, Luis J. Chirosa-Ríos, Emerson Franchini

**Affiliations:** ^1^ Department of Physical Education and Sports, Faculty of Sports Science, University of Granada, Granada, Spain; ^2^ Health Science Faculty, Francisco de Vitoria University, Madrid, Spain; ^3^ Martial Arts and Combat Sports Research Group, Sport Department, School of Physical Education and Sport, University of São Paulo, São Paulo, Brazil

**Keywords:** isometric strength test, grip strength, bilateral deficit, technical gesture, judo and jiu-jitsu

## Abstract

The use of strength training is important for athletes in combat sports such as judo and jiu-jitsu. Specificity is key when prescribing strength training programs for athletes, considering maximal strength, muscular power and strength-endurance. Grappling combat sports require grip control, which is necessary to execute throwing techniques and groundwork. The aim of this study was to verify the bilateral strength deficit in general and in grappling combat sport-specific action in the control group and grapplers. A sample of 73 subjects, divided into 2 groups, was used: the Student Group (15 females and 16 males) and the Grappler Group (29 males and 13 females). The male and female participants attended four laboratory sessions over a 48-hour interval, the first two to familiarize themselves with the Electromechanical Functional Dynamometer (EMFD), and the last two to collect maximal isometric strength data, using a standard grip and a judo/jiujitsu specific grip. Significant differences in mean and peak forces (*p* < 0.001) were found, with high performance combat sport athletes having greater maximal isometric strength compared to students both bilaterally and unilaterally (*p* < 0.001). All comparisons also indicated higher values for males compared to females (*p* < 0.001). The results suggest that specific training in combat sports as well as sex differences play a significant role in maximal isometric strength performance. The type of grip used affects the application of force in the upper limb isometric strength tests, finding a main effect of grip type (*p* < 0.001), with the standard grip obtaining higher levels. However, the dominance between hands remains constant. More research is needed on specific judogi/jiu-jitsu-gi grips and their impact on maximal isometric strength with the EMFD.

## Introduction

Strength training is a key element in the training process for athletes in general (1) and specifically in combat sports (2). When prescribing strength training, several factors need to be considered to optimize the intended results, and one of the most important aspects for athletes is the specificity principle (3). In grappling combat sports such as judo and jiu-jitsu, maximal strength (2), muscle power (4), and strength-endurance (5) are necessary depending on the action to be executed. Studies analyzing the time-motion analysis of these sports (6) provide evidence regarding the specific actions throughout the match and contribute to better training organization. A common element among grappling combat sports is the need for grip control to execute throwing and groundwork techniques, which differ depending on the execution of the grip on a uniform (e.g., judogi, jiu-jitsu-gi) or on the opponent's body (e.g., wrestling). Therefore, the amplitude of the grip and the surface of contact are determined by the characteristics of these sports. As judo was created based on jiu-jitsu techniques, the types of grips are similar in these sports, although the former requires more dynamic strength-endurance due to the predominance of actions in standing combat, whereas isometric strength-endurance seems to be required more during jiu-jitsu matches (7). However, these strength-endurance actions are followed by powerful actions, which are frequently necessary for the successful scoring technique execution (4).

During most scoring actions in judo and jiu-jitsu, both hands are in contact with the opponent's uniform, but each segment executes a different action. For instance, the wrist, elbow, and shoulder angles and force application are distinct, and these aspects are related to side dominance (7, 8). The difference in strength development between sides and the application of force in specific positions may result in a bilateral strength deficit, which occurs when the forces applied simultaneously by two segments are lower than the sum of forces that each segment is able to generate (9–11). A plausible mechanism to explain bilateral strength deficit is that the central nervous system is able to send higher action potentials at different speeds, activating muscle groups in distinct time sequences. During a bilateral task, the central nervous system unifies the energy to release it to a single functional unity (9).

Considering the high prevalence of grip dispute during grappling combat sports matches, and the lapel and sleeve grip positioning necessary to execute most of the throwing techniques (12), as well as ground techniques, a very specific combination of force application is needed when they are executed, resulting in specific muscle activation for the pulling and the lifting actions performed by each side (13). Even though the specific technical actions involved during grip dispute have been extensively analyzed in judo literature [see (8) for a review], and some studies have investigated the strength characteristics of the handgrip of judo and jiu-jitsu athletes (6, 14–17), few studies have considered the force application by different sides (13), and to the best of our knowledge no study has investigated the bilateral strength deficit during actions involving a grip by judo or jiu-jitsu athletes. The analysis of the bilateral strength deficit in these types of tasks involving a general and a sport-specific grip may contribute to a better understanding of this phenomenon (18). Regarding the analysis of the bilateral strength deficit, tests have mainly used actions requiring muscle power or maximal isometric strength. Additionally, when using maximal isometric strength tasks, most researchers used a single specific angle and not a sport-specific gesture (Albalá Gómez, 2016).

Specifically, when considering judo and jiu-jitsu, authors have used several tests, including the countermovement jump test (16, 19, 20), the standing long-jump test (19), the Special Judo Fitness Test (21) and the maximal isometric handgrip test (20, 22, 23). These studies indicated a significant bilateral strength deficit in the lower body, and a better performance when techniques were executed by the dominant side. However, the only article reporting the muscle activation during a judo-specific pulling action (13) did not present the force application on each side, which is quite relevant for this sport. Currently, the use of electromechanical functional dynamometry allows the analysis of force application in specific positions (24, 25). The main advantage of this technique is the high transference and specificity to sports actions. Thus, the main objective of this study was to verify the bilateral strength deficit in general and in grappling combat sport-specific action in the control group and grapplers. The main hypothesis of the present study was that there would be a significant decrease in force production by the athletes when using a specific judogi/jiu-jitsu-gi grip compared with the standard grip condition. Specifically, when performing exercises, mainly pulling exercises, with grips that are automated but not specifically transferable to their sport, they apply a greater amount of force compared to using a specific judogi/jiu-jitsu-gi grip, even though they are performing the same gesture. However, as practically all athletes use standard instruments with common grips or accessories when performing strength training, the difference between specific and standard grips would be lower in athletes compared with the control group. The explanation for this may be the lack of automatism when it comes to applying maximum force in a specific gesture with a specific grip.

## Materials and methods

### Subjects

Seventy-three male and female individuals participated in this study and were divided into subgroups based on their specific characteristics. One of the groups (Student Group) consisted of 16 males and 15 females aged 18–27. The inclusion criteria for the first group were not having any specific combat sports training, and also not having used an EMFD. The second group (Grappler Group) consisted of 29 males and 13 females aged 18–33. The inclusion criteria for the second group were that subjects practiced high-performance combat sports (judo or BJJ) consistently, and also had not used an EMFD.

All participants were informed of the objectives, procedures, and risks of the study and gave their informed written consent before their participation. The study was approved by the Ethics Committee of the University of Granada. It is important to note that all participants had several days to become familiar with the EMFD to avoid any situations that could contaminate the study's data collection on the day of the test. Additionally, each volunteer was instructed not to perform any physical activity immediately before the test sessions.

### Study design

This study compared the strength performance of male and female students and grapplers, specifically Brazilian jiu-jitsu (BJJ) and judo athletes. Each participant attended the laboratory on four different days, with 48-hour intervals, as described below:

- Days 1 and 2: Familiarization session with the Electromechanical Functional Dynamometer (EMFD). The participants became familiar with the equipment by performing three sets of the rowing test (seated, unilateral and bilateral) using the tonic (free weight mode) and incremental mode of the EMFD. On the second day, they performed the isometric rowing test for 6 s.
- Day 3: Data collection of maximal isometric strength. Participants performed the isometric rowing test in a seated position with a standard grip, both unilaterally and bilaterally.
- Day 4: Data collection of maximal isometric strength. Participants performed the isometric rowing test in a seated position with a judo/jiujitsu-specific grip, both unilaterally and bilaterally.

### Procedures

#### Equipment

For the present study, an EMFD (Dynasystem, Model Research, Granada, Spain) was used. This device evaluates various movements through a wide variety of stimuli, including isotonic, isokinetic, elastic, isometric, inertial, eccentric, and vibratory. The nucleus of the machine precisely regulates the angular velocity and the force thanks to its 2,000 W engine. When force is applied to the device cable, which is wound on a roller, it measures the force and linear velocity. The tension produces a signal that is detected by a load cell, which transfers it to a 12-bit resolution analog converter. The displacement and velocity data are collected in a 2,500 ppr (pulse per rotation) encoder connected to the roller.

Two accessories were added to this device to perform different tests. Written instructions on the exercise execution were provided before the warm-up to facilitate the participants' understanding. For the first test, a high pulley grip bar, commonly used for back pull or rowing tasks, was attached to the carabiner. A judo/jiujitsu-specific grip device was attached to the carabiner for the second test.

**Figure 1:**
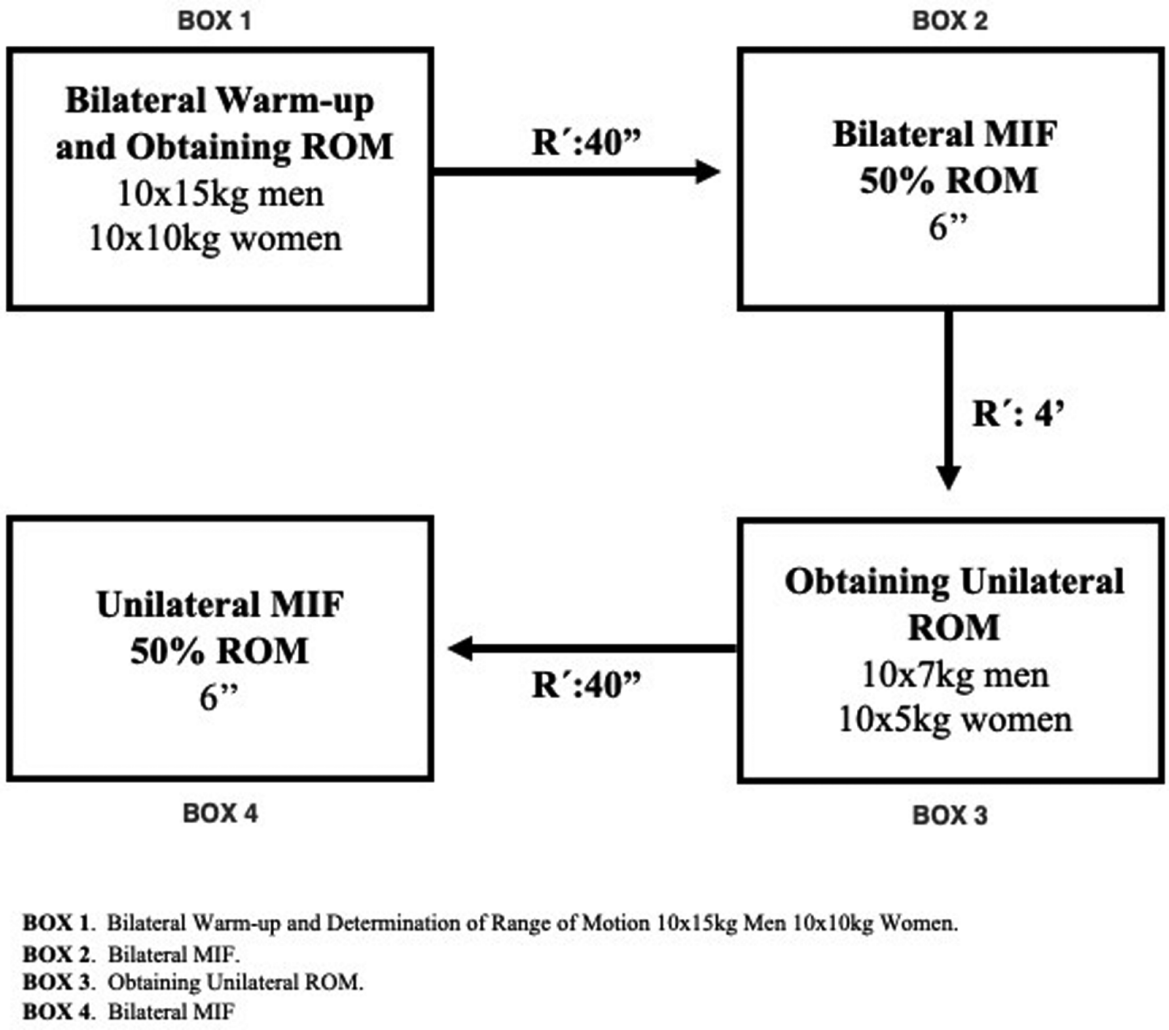
Description of the sequence of steps followed in the evaluation protocol of the MIF test.

For the warm-up, participants performed dynamic rowing gestures on the machine, whereas to determine the variables, they executed the gesture isometrically (FIM test). The isometric test was performed with both the bar grip and the judo/jiujitsu-specific grip device. Both the warm-up and the maximum isometric strength test were performed bilaterally and unilaterally. Both tests had the same implementation protocol (Figure 1).

#### Warm-up and bilateral range of motion measurement

**Figure 2:**
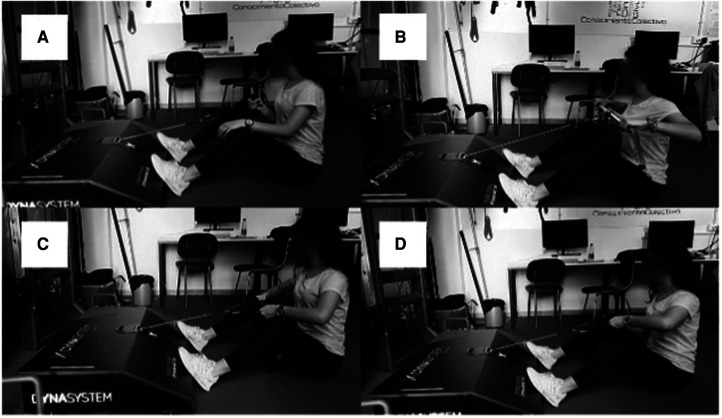
MIF test (50% ROM). (**A**) standard unilateral, (**B**) standard bilateral, (**C**) bilateral with specific grip, (**D**) unilateral with specific grip.

The warm-up began with 8 min of upper-body joint mobility exercises, including shoulder rotation exercises such as adduction-abduction and flexion-extension. After the warm-up, the participants were seated correctly to perform the rowing exercise. Subjects' legs were positioned with knee semi-flexion while placing the soles of the feet on the device surface. The upper extremities held the corresponding grip in pronation, keeping the humerus cubital joint completely extended (Figure 2). Once the participants were in the correct position, they performed 10 controlled bilateral repetitions with no limits on the rope's motion path to provide the complete range of motion (ROM) in the rowing gesture. Females used 10 kg of resistance and males used 15 kg.

#### Bilateral rowing test

Based on the ROM established in the previous 10 repetitions, a 50% ROM was calculated as the most efficient point for performing the Maximum Isometric Strength Test. Once seated at the established distance, the participant performed a bilateral maximum isometric row for 6 (Figure 2). Upon finishing, the participant had a full 4-minute recovery.

#### Obtaining unilateral range of motion

The participant was placed in the same position as in the bilateral test, with their legs outstretched, knee in semi-flexion, and feet soles placed on the dynamometer surface. One of the upper extremities held the corresponding grip in pronation, keeping the humor-cubital joint completely extended. Once the subject was perfectly positioned, they performed 10 controlled unilateral repetitions (5 kg for females and 7 kg for males) with no limits on the cable's ROM to provide the complete ROM in the rowing gesture.

#### Unilateral rowing test

A 50% ROM was calculated for the Maximum Isometric Strength Test based on the ROM established in the previous 10 repetitions. The participant then sat at the established distance and performed a unilateral maximum isometric row for 6 s. After finishing the row with one hand, the participant repeated the same process with the other hand (Figure 2).

### Statistical analysis

The data are presented as mean and standard deviation. Normality was tested using the Shapiro–Wilk test, and the homogeneity of variances was tested using the Levene test. A Student *t* test indicated no difference between the left and right hands for any of the grips (*p* > 0.05). Therefore, hand dominance was not used as a factor in subsequent analysis. For the calculation of bilateral deficit (expressed as a percentage), the formula proposed by Skarabot et al. (26) was used. Standard and specific unilateral (right and left) and bilateral grip peak and mean forces were compared using a two-way analysis of variance (group and gender), followed by an unequal sample size Tukey test. Standard and specific bilateral mean and peak force deficits were compared using a three-way analysis of variance (gender, group, and grip mode) with repeated measurements in the last factor, followed by the Bonferroni test. Sphericity was tested and confirmed via the Mauchly test. The Significance level was set at 5%. Effect sizes were assessed using partial eta squared (*η*_p_^ 2^), using <0.01 (small), <0.06 (medium), and >0.14 (large), respectively (Cohen, 1988). Statistica for Windows (Version 8.0, Stat Soft Inc, Tulsa, United States of America) and Microsoft Excel [version 2013, Microsoft, Remond (WA), United States of America] were used for the analyses.

## Results

Table 1 presents the standard bilateral and unilateral peak and mean grip forces.

**Table 1:** Standard bilateral and unilateral peak and mean grip forces in male and female sport science students and grapplers (values are presented as mean and standard deviation).

	Students		Grapplers	
	Male (*n* = 16)	Female (*n* = 15)	Male (*n* = 29)	Female (*n* = 13)
Bilateral peak force (*N*)^a^	957.5 ± 129.5	580.8 ± 215.6	1,011.4 ± 224.1	649.0 ± 156.8
Bilateral mean force (*N*)^a^	858.2 ± 130.8	518.9 ± 129.5	863.3 ± 188.4	579.4 ± 151.0
Right peak force (*N*)^a^	498.0 ± 85.5	306.6 ± 86.0	498.0 ± 104.3	318.8 ± 57.0
Left peak force (*N*)^a^	483.9 ± 74.8	288.8 ± 79.6	487.3 ± 104.3	363.3 ± 192.4
Right mean force (*N*)^a^	442.1 ± 72.6	275.8 ± 77.8	430.1 ± 92.2	280.8 ± 48.1
Left mean force (*N*)^a^	433.0 ± 67.8	259.0 ± 68.8	422.2 ± 91.2	277.6 ± 39.2

^a^ Main effect of gender (*p* < 0.001), males different from females (*p* < 0.001).

Only an effect of sex was found for the bilateral standard peak force [*F*_1,69_ = 69.36; *p* < 0.001; *η*_p_^ 2^ = 0.50 (large effect)], with higher values for males compared to females (*p* < 0.001). Similarly, an effect of sex was also found for the bilateral mean force [*F*_1,69_ = 63.79; *p* < 0.001; *η*_p_^ 2^ = 0.48 (large effect)], with higher values for males compared with females (*p* < 0.001).

For unilateral standard forces, only an effect of sex was found for right peak force [*F*_1,69_ = 70.95; *p* < 0.001; *η*_p_^ 2^ = 0.51 (large effect)], left peak force [*F*_1,69_ = 31.72; *p* < 0.001; *η*_p_^ 2^ = 0.32 (large effect)], right mean force [*F*_1,69_ = 67.70; *p* < 0.001; *η*_p_^ 2^ = 0.51 (large effect)], and left mean force [*F*_1,69_ = 76.09; *p* < 0.001; *η*_p_^ 2^ = 0.52 (large effect)]. All post-hoc comparisons indicated higher values for males compared to females (*p* < 0.001).

Table 2 presents the specific bilateral and unilateral peak and mean grip forces.

**Table 2:** Specific bilateral and unilateral peak and mean grip forces in male and female sport science students and grapplers (values are presented as mean and standard deviation).

	Students		Grapplers	
	Male (*n* = 16)	Female (*n* = 15)	Male (*n* = 29)	Female (*n* = 13)
Bilateral peak force (*N*)^a^	800.9 ± 122.2	517.4 ± 159.7	826.2 ± 233.2	492.9 ± 145.0
Bilateral mean force (*N*)^a^	726.6 ± 120.9	470.4 ± 144.1	720.5 ± 201.7	450.6 ± 144.1
Right peak force (*N*)^a^	426.5 ± 68.6	284.3 ± 88.2	443.9 ± 127.9	300.3 ± 52.4
Left peak force (*N*)^a^	407.2 ± 52.9	282.2 ± 88.9	454.7 ± 118.1	303.8 ± 53.4
Right mean force (*N*)^a^	377.8 ± 61.7	250.3 ± 53.5	378.5 ± 106.1	264.6 ± 52.4
Left mean force (*N*)^a^	362.9 ± 55.9	252.5 ± 67.1	393.7 ± 91.1	272.9 ± 45.9

^a^ Main effect of gender (*p* < 0.001), males different from females (*p* < 0.001).

For the bilateral specific peak force only a main effect of sex was found [*F*_1,69_ = 46.24; *p* < 0.001; *η*_p_^ 2^ = 0.40 (large effect)], with higher values for males compared to females (*p* < 0.001). An effect of sex was also found for the bilateral mean force [*F*_1,69_ = 41.89; *p* < 0.001; *η*_p_^ 2^ = 0.38 (large effect)], with higher values for males compared to females (*p* < 0.001).

For unilateral specific forces, only an effect of sex was found for right peak force [*F*_1,69_ = 41.23; *p* < 0.001; *η*_p_^ 2^ = 0.37 (large effect)], left peak force [*F*_1,69_ = 43.48; *p* < 0.001; *η*_p_^ 2^ = 0.39 (large effect)], right mean force [*F*_1,69_ = 38.19; *p* < 0.001; *η*_p_^ 2^ = 0.36 (large effect)], and left mean force [*F*_1,69_ = 41.99; *p* < 0.001; *η*_p_^ 2^ = 0.38 (large effect)]. All post-hoc comparisons indicated higher values for males compared to females (*p* < 0.001).

Table 3 presents the bilateral peak and mean grip force deficits.

**Table 3:** Standard and specific bilateral peak and mean grip force deficits in male and female sport science students and grapplers (values are presented as mean and standard deviation).

	Students		Grapplers	
	Male (*n* = 16)	Female (*n* = 15)	Male (*n* = 29)	Female (*n* = 13)
Standard bilateral peak force deficit (%)^a–g^	−1.7 ± 7.4	−1.2 ± 12.4	3.6 ± 14.7	9.9 ± 26.6
Specific bilateral peak force deficit (%)	−4.1 ± 6.4	−8.1 ± 11.3	−7.4 ± 16.7	−19.8 ± 13.1
Standard bilateral mean force deficit (%)^a,h,i^	−1.5 ± 8.3	−2.1 ± 11.3	2.0 ± 15.1	3.8 ± 20.6
Specific bilateral mean force deficit (%)	−1.8 ± 9.3	−6.1 ± 12.9	−5.5 ± 17.6	−17.6 ± 15.3

^a^ Grip mode main effect (*p* < 0.001), higher values in standard compared with specific grip (*p* < 0.001).

^b^ Grip mode and gender interaction effect (*p* < 0.05), higher values for males in the standard grip compared to females in the specific grip (*p* < 0.001).

^c^ Grip mode and gender interaction effect (*p* < 0.05), males in the specific grip presented higher values compared to females in the standard grip (*p* = 0.028).

^d^ Grip mode and gender interaction effect (*p* < 0.05), females in the specific mode presented lower deficits compared to females in the standard mode (*p* < 0.001).

^e^ Grip mode and group interaction effect (*p* < 0.05), students using standard grip achieved higher deficits than athletes using specific grip (*p* = 0.034).

^f^ Grip mode and group interaction effect (*p* < 0.05), athletes using standard grip achieved higher deficits than students using specific grip (*p* = 0.007).

^g^ Grip mode and group interaction effect (*p* < 0.05), athletes using standard grip achieved higher deficits than athletes using specific grip (*p* < 0.001).

^h^ Grip mode and gender interaction effect (*p* < 0.05), higher values for males in the standard grip compared with females in the specific grip (*p* < 0.01).

^i^ Grip mode and gender interaction effect (*p* < 0.05), higher values for females in the standard grip compared to females in the specific grip (*p* < 0.01).

For the bilateral peak grip force deficit, a main effect of grip mode was found [*F*_1,69_ = 26.62; *p* < 0.001; *η*_p_^ 2^ = 0.28 (large effect)], with higher values for standard grip compared with specific grip (*p* < 0.001). An interaction was found between grip mode and sex [*F*_1,69_ = 5.77; *p* = 0.019; *η*_p_^ 2^ = 0.08 (large effect)]. Males presented a higher deficit in the standard grip compared to the specific grip (*p* = 0.053). Higher values were found for males in the standard grip compared to females in the specific grip (*p* < 0.001), and males in the specific grip presented lower values compared to females in the standard grip (*p* = 0.028). Additionally, females in the specific mode presented lower deficits compared to females in the standard grip (*p* < 0.001). An interaction was found between grip mode and group [*F*_1,69_ = 10.52; *p* = 0.002; *η*_p_^ 2^ = 0.13 (large effect)]. Students using standard grip achieved higher deficits than athletes using specific grip (*p* = 0.034). Athletes using a standard grip achieved higher deficits than students (*p* = 0.007) and athletes (*p* < 0.001) using the specific grip.

For the bilateral mean grip force deficit, a main effect of grip mode was found [*F*_1,69_ = 16.02; *p* = 0.001; *η*_p_^ 2^ = 0.19 (large effect)], with higher values for standard grip compared with specific grip (*p* < 0.001). An interaction was found between grip mode and sex [*F*_1,69_ = 4.46; *p* = 0.038; *η*_p_^ 2^ = 0.06 (large effect)]. Females using the specific grip achieved lower values compared to males (*p* = 0.004) and females (*p* = 0.002) using the standard grip. An interaction was found between grip mode and group [*F*_1,69_ = 8.71; *p* = 0.004; *η*_p_^ 2^ = 0.11 (large effect)], with grapplers using a standard grip achieving higher values compared with the specific grip (*p* = 0.002).

## Discussion

The main hypothesis of the present study was that there would be a significant but lower decrease in force production by the athletes when using a specific judogi/jiu-jitsu-gi grip compared to the standard grip condition for the control group. This was partially confirmed as the specific grip resulted in lower deficits compared to the standard grip, and the deficit in this condition was higher in male grapplers compared with females and students. Moreover, the peak and mean forces were higher in males than in females for both unilateral and bilateral modes, using standard and specific grips.

The values observed in females were around 60%–70% of those measured in their male counterparts, which is within the typical range of differences reported between sexes (27). However, when male and female judo athletes were compared, Franchini et al. (27) indicated that high-level male and female judo athletes differed less in relative maximal isometric handgrip strength when compared to lower-level male and female judo athletes. These authors hypothesized that this was likely due to the intensive training undergone by athletes of both sexes, a factor that could decrease the difference in relative strength.

The fact that grapplers did not differ from students is supported by the fact that judo athletes' maximal isometric handgrip strength was classified as good when compared to North American judo athletes (27). Indeed, it has been suggested that during judo and BJJ matches, athletes rarely exert their maximal isometric strength (28, 29). Instead, they constantly apply an elevated level of force to maintain their grip, suggesting that strength-endurance may be more important for both judo and BJJ athletes than maximal isometric strength (5, 28).

The values of the bilateral deficit observed in the present study were similar to those reported in judo athletes using the maximal isometric handgrip strength test (−0.9% to −4.5%) (23), but lower than those reported using the same task after two judo matches (∼10%) (19), and much lower than those found in parameters measured in the countermovement jump (CMJ; 20%–31%) (20). Therefore, it seems that when a more powerful action (i.e., CMJ) is performed or when fatigue has set in, the bilateral deficit is accentuated. However, maximal strength in controlled conditions was similar to that found in our study.

Regarding the bilateral deficit findings, since the specific grip involves grasping the kimono fabric, the main limiting factor is the small muscle groups involved in the finger-gripping action. Thus, a smaller variation would be observed in these groups compared to bigger muscle groups that are more frequently recruited in daily tasks and resistance training exercises. However, it is important to consider that grapplers presented an even smaller bilateral deficit in the specific grip condition than students in the standard condition. In contrast to Turnes et al. (23), who found a handgrip isometric bilateral deficit only in advanced judo athletes, our study revealed different results.

It is likely that the lower deficit in grapplers using the specific grip compared to students using the standard grip can be explained by the specificity principle. Specifically, the constant use of the specific grip and the need to develop strong finger muscles to control the opponent during judo and BJJ may have resulted in well-trained muscles and balanced force between sides for these athletes. Conversely, inexperienced individuals may have a higher variation in the muscle groups responsible for the gripping action in the kimono. Additionally, it is also important to note that sexes differed only when interacting with the grip mode, which may be related to the higher variation in training experience in females compared to males due to the frequent discrimination of females in combat sports.

## Conclusion

In conclusion, the results of this study indicate that the type of grip used in upper limb isometric strength tests can significantly affect the application of force. The dominant hand was found to be stronger than the non-dominant hand in both types of grips. However, the type of grip used did not have a significant impact on the amount of force produced. This suggests that either grip can be used during the specific gesture being performed while maintaining the same force output. Furthermore, the study found that the type of grip used can influence the application of maximal isometric force in specific technical gestures in both populations. The hypothesis that there would be a significant but lower decrease in force generated by athletes when using a specific judogi/jiu-jitsu-gi grip, in comparison with the standard grip for the control group, was partially confirmed. Lower deficits were observed for the specific grip compared to the standard grip. Additionally, the deficit in this condition was higher for male grapplers when compared with females and students. The lack of research using judogi/jiu-jitsu-gi specific grips to evaluate maximal isometric strength with the EMFD means that the results of this study may be limited. It is possible that the lack of automation when performing common pulling gestures using a specific grip could have influenced the results. Therefore, future studies should investigate the effects of different types of grips on maximal isometric strength and how these findings can be applied to specific sports or activities. Additionally, research on the effects of training and conditioning on grip strength could be valuable in improving athletic performance and injury prevention.

## Data Availability

The raw data supporting the conclusions of this article will be made available by the authors, without undue reservation.
